# Does treatment of intestinal helminth infections influence malaria? Background and methodology of a longitudinal study of clinical, parasitological and immunological parameters in Nangapanda, Flores, Indonesia (ImmunoSPIN Study)

**DOI:** 10.1186/1471-2334-10-77

**Published:** 2010-03-25

**Authors:** Aprilianto E Wiria, Margaretta A Prasetyani, Firdaus Hamid, Linda J Wammes, Bertrand Lell, Iwan Ariawan, Hae Won Uh, Heri Wibowo, Yenny Djuardi, Sitti Wahyuni, Inge Sutanto, Linda May, Adrian JF Luty, Jaco J Verweij, Erliyani Sartono, Maria Yazdanbakhsh, Taniawati Supali

**Affiliations:** 1 Department of Parasitology, Faculty of Medicine, University of Indonesia, Jakarta, Indonesia; 2 Department of Parasitology, Leiden University Medical Center, Leiden, The Netherlands; 3 Department of Microbiology, Faculty of Medicine, Hasanuddin University, Makassar, Indonesia; 4 Medical Research Unit, Albert Schweitzer Hospital, Lambaréné, Gabon; Department of Parasitology, Institute of Tropical Medicine, University of Tübingen, Tübingen, Germany; 5 Department of Biostatistics, School of of Public Health, University of Indonesia, Jakarta, Indonesia; 6 Department of Biostatistics, Leiden University Medical Centre, Leiden, The Netherlands; 7 Department of Parasitology, Faculty of Medicine, Hasanuddin University, Makassar, Indonesia; 8 Department of Medical Microbiology, Radboud University Nijmegen Medical Centre, Nijmegen, The Netherlands

## Abstract

**Background:**

Given that helminth infections are thought to have strong immunomodulatory activity, the question whether helminth infections might affect responses to malaria antigens needs to be addressed. Different cross-sectional studies using diverse methodologies have reported that helminth infections might either exacerbate or reduce the severity of malaria attacks. The same discrepancies have been reported for parasitemia.

**Methods/Design:**

To determine the effect of geohelminth infections and their treatment on malaria infection and disease outcome, as well as on immunological parameters, the area of Nangapanda on Flores Island, Indonesia, where malaria and helminth parasites are co-endemic was selected for a longitudinal study. Here a Double-blind randomized trial will be performed, incorporating repeated treatment with albendazole (400 mg) or placebo at three monthly intervals. Household characteristic data, anthropometry, the presence of intestinal helminth and *Plasmodium spp *infections, and the incidence of malaria episodes are recorded. *In vitro *cultures of whole blood, stimulated with a number of antigens, mitogens and toll like receptor ligands provide relevant immunological parameters at baseline and following 1 and 2 years of treatment rounds. The primary outcome of the study is the prevalence of *Plasmodium falciparum *and *P. vivax *infection. The secondary outcome will be incidence and severity of malaria episodes detected via both passive and active follow-up. The tertiary outcome is the inflammatory cytokine profile in response to parasite antigens. The project also facilitates the transfer of state of the art methodologies and technologies, molecular diagnosis of parasitic diseases, immunology and epidemiology from Europe to Indonesia.

**Discussion:**

The study will provide data on the effect of helminth infections on malaria. It will also give information on anthelminthic treatment efficacy and effectiveness and could help develop evidence-based policymaking.

**Trial registration:**

This study was approved by The Ethical Committee of Faculty of Medicine, University of Indonesia, ref:194/PT02.FK/Etik/2006 and has been filed by ethics committee of the Leiden University Medical Center. Clinical trial number:ISRCTN83830814. The study is reported in accordance with the CONSORT guidelines for cluster-randomized studies.

## Background

Worldwide, more than a billion people are infected by geohelminths, with a majority harboring roundworms (*Ascaris lumbricoides*), whipworms (*Trichuris trichiura*), and/or hookworms (*Necator americanus *or *Ancylostoma duodenale*) [1-3]. Such helminths modify the immune system to induce predominant production of T-helper-2 (Th2) cytokines (interleukin/IL-4, IL-5, IL-9, IL-10, IL-13), associated with increased levels of immunoglobulin E (IgE) and eosinophilia [4,5]. Another hallmark of chronic helminth infections is their ability to induce a strong regulatory network characterized by T cell hyporesponsiveness and the increased production of suppressive cytokines such as IL-10 and TGF-β [6]. Their ability to induce regulatory responses is thought to be advantageous for both the parasite and the host as it allows the survival of the parasite for extended periods of time within the host while preventing overt pathological reactions that would otherwise be damaging to the host [7]. The latter may explain why such helminth infections rarely result in overt clinical manifestations.

Although the immunological consequences of helminth infections primarily reflect responses directed towards helminth antigens, there may be spill-over effects on responses to unrelated antigens [6,8]. Whereas the marked Th2 polarization may compete with Th1 cytokines to affect the magnitude of a Th1 response to an incoming antigen, a strong regulatory response can dampen immune reactivity of both Th1 and Th2 types to a third party antigen. Not surprisingly, then, it is argued that helminth infections might modulate the human immune response to common co-infections such as malaria, TB and HIV/AIDS [9-11].

Malaria itself is one of the most serious infectious diseases, infecting 5-10% of the world's population, with 300-600 million cases and more than 2 million deaths annually [12]. In areas of high malaria transmission, the burden of disease is borne by infants and young children [13,14], whilst, in areas of lower transmission, primary infection might also occur later in life, causing severe illness [12,15,16].

Co-infection is the norm in nature [9,17], since helminth infections of different species are often endemic in the same communities that are exposed to infection with plasmodia [18]. The question of whether helminth infections affect the course of malaria has been addressed in various reports [19] in the past few years. Only a small number of papers report on population studies and most of them were cross-sectional in nature, which in contrast to longitudinal studies might not be able to demonstrate the actual dynamics of infection. An early study by Murray and colleagues (1977) in a malnourished population at Anjouan, Comoros Islands suggest that *A. lumbricoides *might suppress malaria symptoms [20]. Since then studies of co-infections have shown helminths to either exacerbate [21] or reduce [22-24] the severity of malaria. The reasons for conflicting data on the effect of helminth co-infection on malaria disease outcomes could be due to differences in study design, study groups, and possibly, most importantly, the helminth species investigated [18,25].

Although helminth infections are considered as pathogens, it is clear that their course of infection is relatively free of overt clinical manifestations and in recent years several reports have shown their suppressive effect on diseases such as allergy [3,26], autoimmunity [27], and inflammatory bowel disease [28], highlighting a possible beneficial effect on inflammation [29]. To assess the influence of helminth infections on inflammatory diseases taking into account environmental and genetic influences in a longitudinal setting, the ImmunoSPIN project http://www.immunospin.org has been initiated. As part of the project ImmunoSPIN, the helminth-malaria sub project (ImmunoSPIN-Malaria) was designed to determine whether and how helminth infections may affect the course of a malaria infection. This study is a double-blind randomized trial of placebo and anthelminthic treatment to elucidate the impact of helminth infections on malaria longitudinally in an area where helminth infections and malaria coexist. It is expected to provide new information and contribute to our understanding of how regulatory responses that may be induced by chronic helminth infections affect inflammatory conditions, especially on malaria infection.

This paper presents the rationale of the ImmunoSPIN-Malaria study that, at the clinical and biological level, aims to discern plausible and meaningful interactions between these infections.

## Methods/Design

### Study area and population

**Figure 1:**
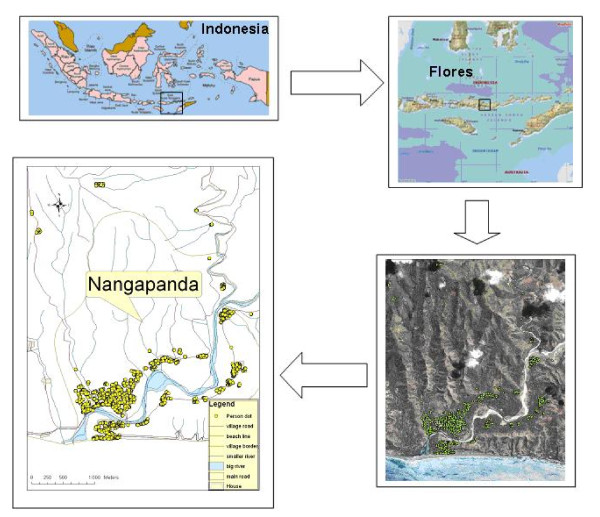
**The map of the study area in Nangapanda, Flores, Indonesia**.

Nangapanda is a sub-district of the Ende district, Flores Island, Indonesia and is situated in a coastal area with a population of about 22000 (Figure 1). Situated near the equator (8°45'S, 121°40'E)[30], it is characterized by a uniform high temperature, in the range of 23-33.5°C, with humidity of 86-95%. Average yearly rain fall is 1.822 mm with about 82 rainy days, especially from November to April, with the peak in December until March. Malaria is reported to be highly prevalent in this area [31]. Preliminary surveys conducted in 2005 and 2006 found this area to be endemic for geohelminths (*A. lumbricoides*, hookworms and *T. trichiura*) and malaria parasites (*P. falciparum *and *P. vivax*). The sub-district is divided into villages of which those located near the primary health centre (Puskesmas), Ndeturea, Ndorurea1, and Ndorurea are the focus of ImmunoSPIN-Malaria study.

The majority in the study area is Ende tribe and migrants from neighbouring district of Bajawa that have populated the island for more than 300 years. Most individuals work as farmers and grow their own food. Recently also government employees and individuals owning small businesses moved to the area. Individuals with Chinese and Arabic ethnicity have come to the area about 200 years ago and most of them, run business corporations with other tribes. There are very few other tribes from outside of Flores and most of them were brought from outside by marriage with Flores natives. The Nangapanda population is dynamic and individuals shift address within the area as well as move in and out of the area for purposes of studying, marriage, or searching for jobs.

### Study design, data and sample collection

Baseline mapping of houses by Global Positioning System (GPS) system has allowed maps to be generated using ArcGIS 9,1 software (ESRI, USA). In order to perform spatial analyses based on individual data, geographical coordinates were assigned to each individual as well as centroid coordinates to households.

Community workers were recruited and trained to do questionnaires, follow-ups, malaria surveys (finger prick), and distribute treatment or placebo, as well as doing health promotion among the population. Each community worker will be in charge of a certain number of households that they visit monthly for recording movement and active follow-up of malaria symptoms, and three monthly for finger pricking and distributing the treatment.

The study is designed as a double-blind randomized trial with two arms. One arm is treatment with albendazole (single dose of 400 mg) [32], while the other arm is treatment with matching placebo (both tablets from PT Indofarma Pharmaceutical, Bandung, Indonesia). The treatment is provided every three months for a period of two years (a total of 8 treatments) to everyone, except children below two years of age, pregnant women or severely ill persons. Computer aided block randomization by household, using Random Allocation Software [33] will be assigned to the treated and placebo groups. The treatment is coded as A or B and the codes are concealed from investigators and patients. Labels with the study subject ID are printed from a computer database and attached to the appropriate strip of treatment by a separate team located in Jakarta without the involvement of the study investigators. An interim analysis will be performed by the monitoring committee, 1 year after treatment to test for any adverse effects that retention of anthelminthic treatment might have on the growth of children and on the incidence of malaria episodes (in contrast to the hypothesis being tested). If the trial continues, the final unblinding of the codes will take place two years after treatment.

On approval of individuals in the area, peripheral blood will be collected from a subset of individuals over 4 years of age that were randomly selected for whole blood culture based on households. The assay will be done at baseline, 1 and 2 years after treatment. For those who are not included in whole blood assays, malaria evaluation by finger pricking will be obtained aiming to include the whole population in the study area.

Every household receives a card identifying all family members. This card is used when community workers visit homes and when family members visit the Puskesmas. Data on socioeconomic status and health status is collected at baseline and 2 years after treatment using questionnaires in Bahasa Indonesia. Monthly data such as birth, death and migration will be recorded. Newborns and individuals who enter the study area will be registered with a newly assigned ID number as well as their geographical coordinates. Active follow-up includes a questionnaire with questions on clinical complaints in general and clinical malaria in particular and will be conducted monthly by community workers. In addition to the questionnaire, blood slides will be collected at three monthly intervals to test for plasmodia. For examination of intestinal helminths, stool samples will be collected yearly. Samples will be used for microscopy and PCR analyses. Passive follow-up will be held in collaboration with the Puskesmas that keeps clinical records of individuals that visit either for consultation or for overnight admission, including information on diagnosis and treatment. If malaria is suspected, two blood slides will be collected: one to be examined by the Puskesmas staff and another for re-examination by the research team in Jakarta at the Department of Parasitology, University of Indonesia (UI).

All blood samples (serum, cell pellet, plasma, and whole blood), blood culture supernatants, as well as stool samples for PCR, will be kept at -20°C in a temperature recorded freezer which is checked twice a day and will be sent to Jakarta on dry ice for storage -20°C or -80°C.

### Outcomes and case definitions

The study aims to determine whether and how helminth infections may affect the course of infection with malaria parasites and disease outcome. Therefore we will monitor disease in individuals treated with albendazole compared to placebo. The primary outcome is the prevalence of infection with *P. falciparum *and *P. vivax *up to 2 years after treatment, the secondary outcome is the incidence and severity of malaria recorded as a result of passive and active follow-up up to 2 years after treatment. The tertiary outcome is the inflammatory cytokine responses to *P. falciparum *antigens 1 and 2 years after treatment.

Helminth and plasmodia infections will be defined by the presence of parasites detected by microscopic examination of stool and blood samples respectively and will be confirmed by molecular (PCR-based) methods [34-38]. A malaria case is defined as an individual with typical malaria symptoms and a blood slide containing plasmodia asexual forms. Typical malaria case definitions: fever (oral temperature ≥ 37.5°C) and/or history of fever in the past 48 hours with a positive slide (*P. vivax *≥ 250/ul, *P. falciparum *≥ 1000/ul). Asymptomatic carriers have a blood slide positive for *P. falciparum *and/or *P. vivax *asexual forms, as well as positive result by PCR and no concomitant clinical symptoms. Suspected malaria cases are defined as individuals who report symptoms typical of malaria but for whom no slide is available at the time of presentation with symptoms.

### Parasitological examination

#### Stool examination by microscopy

The Harada Mori method will be carried out on fresh stool samples to detect hookworm larvae. A certain amount of each stool sample is preserved in formalin (4%) and kept at room temperature for microscopic examination. The formol-ether acetate concentration method [39] is performed on the formalin preserved stool samples followed by microscopical examination for intestinal helminth infections, as well as protozoan infections. For hookworm detection, an amount of fresh stool sample is incubated using filter paper soaked by distilled water inside sealed plastic tubes according to the Harada Mori method [40].

#### Stool examination by real-time PCR

For DNA isolation from stool, approximately 100 mg unpreserved faeces (that was kept at -20°C) are suspended in 200 μl PBS containing 2% polyvinylpolypyrolidone (PVPP; Sigma, Steinheim, Germany). DNA isolation and setup of the PCR reactions are performed using a custom-made Hamilton robot platform (made in Germany). After heating for 10 min at 100°C suspensions are treated with sodium dodecylsulphate-proteinase K for 2 h at 55°C. DNA is isolated using QIAamp DNA-easy 96-well plates (QIAgen, Venlo, The Netherlands) [36]. Within the isolation lysis buffer, 10^3 ^PFU/ml Phocine herpes virus 1 (PhHV-1) is added to serve as an internal control [41].

#### *A. duodenale, N. americanus *(hookworm), *A. lumbricoides, S. stercoralis *real-time PCR (ANAS-PCR) [or just "helminth rt-PCR"]

Sequences of the *A. duodenale-*, *N. americanus-*, and *S. stercoralis*-specific primers and probes are used as described previously [37,38] with some modifications in fluorophore- and quencher-chemistry. Minor groove binding (MGB) probes are replaced by XS probes (Biolegio, Malden, The Netherlands) and to accommodate the specific fluorophor combination of the CFX real-time PCR system (Bio-Rad laboratories, USA) the *A. duodenale *specific XS-probe is labelled with Texas Red and the *S. stercoralis*-specific probe is labelled with Quasar-705. The *A. lumbricoides*-specific primers and probe are chosen using Primer Express software (Applied Biosystems, Foster City, CA), from the internal transcribed-spacer-1 (ITS1) sequence of *A. lumbricoides *(GenBank accession ALJ000895). The *A. lumbricoides*-specific primers, Alum96F 5'-GTAATAGCAGTCGGCGGTTTCTT-3' and Alum183R 5'-GCCCAACATGCCACCTATTC-3' amplify an 87-bp fragment of the ITS1 sequence and the XS-probe Alum124T Yakima Yellow-5'-TTGGCGGACAATTGCATGCGAT-3'-XS is used to detect the *A. lumbricoides*-specific product. The real-time PCRs were optimized first as monoplex assays with 10-fold dilution series of *A. duodenale*, *N. americanus*, *A. lumbricoides*, and *S. stercoralis *DNA, respectively. The monoplex real-time PCRs were thereafter compared with the multiplex PCR with the PhHV internal control. The cycle threshold (Ct) values obtained from testing the dilution series of each pathogen in both the individual assay and the multiplex assay were similar, and the same analytical sensitivity was achieved. The multiplex ANAS PCR showed 100% specificity when tested against 145 DNA controls derived from a wide range of intestinal microorganisms [35].

Amplification reactions are performed in white PCR plates in a volume of 25 μl with PCR buffer (HotstarTaq master mix, QIAgen, Germany), 5 mM MgCl_2, _2.5 μgram Bovine Serum Albumin (Roche Diagnostics Nederland B.V., Almere, the Netherlands), 5 pmol of each *A. duodenale-*specific primer, and of each *N. americanus*-specific primer, 2 pmol of each *A. lumbricoides*-specific primer, 2.5 pmol of each *S. stercoralis*-specific primer and 3.75 pmol of each PhHV-1-specific primer, 1.25 pmol of each *N. americanus*-specific XS-probe, *A. lumbricoides-*specific XS-probe, *S. stercoralis-*specific double-labelled probe, and PhHV-1-specific double-labelled probe, and 2.5 pmol of the *A. duodenale-*specific XS-probe, and 5 μl of the DNA sample.

Amplification consists of 15 min at 95°C followed by 50 cycles of 15 s at 95°C, 30 s at 60°C, and 30 s at 72°C. Amplification, detection, and analysis are performed with the CFX real-time detection system (Bio-Rad laboratories). The PCR output from this system consists of a cycle-threshold (Ct) value, representing the amplification cycle in which the level of fluorescent signal exceeds the background fluorescence, and reflecting the parasite-specific DNA load in the sample tested. Negative and positive control samples are included in each amplification run.

The amplification is considered to be hampered by faecal inhibitory factors if the expected cycle threshold (Ct) value of 33 in the PhHV-specific PCR is increased by more than 3.3 cycles.

#### Blood examination by microscopy

To detect *P. falciparum, P. vivax, P. malariae*, and *P. ovale*, blood slides (thick and thin) are stained with Giemsa [42] followed by microscopic examination [43].

#### Blood examination by real-time PCR

DNA was isolated from 200 μl blood with QIAamp DNA-easy 96-well plates according to the manufacturer's recommendations. DNA isolation and setup of the PCR reactions are performed using a custom-made Hamilton robot platform.

#### *P. falciparum, P. vivax, P. ovale*, and *P. malariae *(malaria) real-time PCR

Sequences of the *Plasmodium-*specific primers and the *P. falciparum, P. vivax, P. ovale, and P. malariae*-specific probes are used as described previously[34,44] with some modifications in the fluorophore- and quencher-chemistry. Minor groove binding (MGB) probes are replaced by XS probes and to accommodate the specific fluorophor combination of the CFX system the *P. falciparum*-specific XS-probe is labelled with Yakima Yellow, the *P. ovale-*specific XS-probe is labelled with Texas Red and the *P. malariae-*specific probe is labelled with Quasar-705. An additional *P. ovale*-type 2 XS-probe (Texas Red 5'-TCCAAAAGGAATTTTCTTATT-3'-XSQ) is used for sensitive detection of the *P. ovale *genetic variant type 2 (GenBank accession X99790/J001527).

Amplification reactions of each DNA sample are performed in white PCR plates, in a volume of 25 μl with PCR buffer (HotstarTaq master mix), 5 mmol/l MgCl2, 12.5 pmol of each Plasmodium-specific primer and 15 pmol of each PhHV-1-specific primer, 1.5 pmol of each *P. falciparum, P. vivax-, P. malariae*-specific XS-probes, and PhHV-1-specific Cy5 double-labelled detection probe, and 2.5 pmol of each *P. ovale*-specific XS-probes (Biolegio), and 5 μl of the DNA sample were used.

Amplification, detection, and analysis are performed as described for the faecal PCR.

### Immunological Measurements

#### Whole blood culture and cytokine measurements

Blood samples (6 ml) are drawn into Vacutainers (BD, Franklin Lakes, NJ, USA) containing sodium heparin as anticoagulant. Within 6 hours, blood cultures are set up according to methods that were optimized and tested under field conditions during pilot studies. The heparinized blood is diluted 1:4 with RPMI 1640 medium (Invitrogen, Breda, The Netherlands) (supplemented with 2 mM glutamate, 1 mM pyruvate, 100 IU penicillin and 100 μg/ml streptomycin) and cultured in 96 well round bottomed plates in 37°C with 5% CO_2_. Stimulations were performed with medium/control, PHA (2 μg/ml, Wellcome Diagnostics, Darford, UK), LPS (1 ng/ml Sigma-Aldrich, Zwijndrecht, The Netherlands), Pam3Cys (100 ng/ml, Cayla-InvivoGen Europe, Toulouse, France), PolyIC (50 μg/ml, Cayla-InvivoGen Europe, Toulouse, France), *Ascaris *antigen (20 μg/ml, as prepared by van Riet E et al [45]), iRBC (1 × 10^6^, prepared by Radboud University Nijmegen Medical Centre, Nijmegen, The Netherlands[46]), uRBC (1 × 10^6^, prepared by Radboud University Nijmegen Medical Centre, Nijmegen, The Netherlands [46]). Supernatants are collected on day 1 (unstimulated control, LPS, Pam3Cys) and day 3 (unstimulated control, PHA, Ascaris, iRBC, uRBC, PolyIC). Cytokine concentrations in supernatants are assessed by means of immunobead-based multiplex assays. This is an assay that permits simultaneous quantification of multiple cytokines in a small sample volume. Panels of capture antibody-coated beads and labeled detection antibodies are purchased from Bio Source (Camarillo, California, USA). The cytokines measured will represent pro- and anti-inflammatory, Th1 and Th2 cytokines: TNF-α and IL-10 from day 1 supernatants as well as IL-2, IL-5, IL-10, IFN-γ, and TNF-α for day 3 supernatants. Analysis will be performed on a Liquichip 200^® ^Workstation (Qiagen, Venlo, The Netherlands) using Liquichip analyzer software (Qiagen, Venlo, The Netherlands).

#### Total IgE

Total IgE will be measured as described previously [47]. Briefly, maxisorp plates (Thermo Fisher Scientific, Roskilde, Denmark) are coated overnight with 100 μl/well rabbit anti-human IgE (Dako, Glostrup, Denmark) at 1/1400 dilution in 0.1 M carbonate buffer. Plates are blocked with 100 μl/well phosphate buffered saline (PBS) containing 5% bovine serum albumin (BSA, Albumin Fraction V, Boehringer, Mannheim, Germany). Sera to be tested are diluted 1:200 in PBS containing 5% fetal calf serum (FCS, Greiner Bio-One, Alphen a/d Rijn, Netherlands). A positive standard serum containing human IgE (NIBSC, Potters Bar, UK) is diluted down 1/3 in a series from 90 IU/ml until a final concentration 0.12 IU/ml on each plate and incubated for 1 hour at room temperature. After washing step, IgE biotinylated goat anti-human IgE antibody (1/1000 (Vector Laboratories, Burlingame, CA, USA)) is added followed by Streptavidin Alkaline Phosphatase conjugate (1/3000 (Boehringer, Mannheim, Germany)). The colour is developed by addition of para-nitrophenylphosphate substrate (p-NPP (Boehringer, Mannheim, Germany)) diluted in diethanolamine buffer (DEA, 0.5 mM Mg CL2, 0.1 M DEA, pH 9.6 (Merck, Darmstadt, Germany) and optical density is measured at 405 nm.

### Statistical analyses

A database using MS Access is developed for this study. At baseline, we will analyze whether helminth infected subjects are at a higher risk of having *P. falciparum *or *P. vivax *infections in terms of presence of infection and intensity. The effect of anthelminthic treatment will be assessed 1 and 2 years after treatment by analyzing the prevalence ratios as well as the incidence ratios of malaria infection (clinical cases and parasitemia). In addition, treatment will be compared with placebo for reduction of helminth infection and is based on an intention to treat principle, in anticipation of individual movement between treatment groups. Cytokine profiles in response to parasite antigens will be analyzed for levels of pro- and anti-inflammatory cytokines.

The characterization of immune responses to helminth infections, malaria infections and co-infections will be assessed prior to treatment using linear regression [48]. As cytokine levels are non-normally distributed, we will use log-transformed cytokine data for all analyses regarding effect of helminth treatment on cytokine profile and susceptibility to plasmodium infection. For these analyses, multilevel modeling will be used and the use of longitudinal data will take into account repeated measurements [48]. Any bias related to selection of participants and outcome of treatment will be assessed by comparing individuals that are lost to follow-up and individuals that are not lost and will be compared on the basis of their baseline characteristics, age, gender, village, and socioeconomic status and parasitic infections. A similar assessment will also be undertaken to compare the characteristics of individuals in the treatment and placebo groups at inclusion into the study. Chi-square analyses will be used to test proportions.

Multiple regression analysis will be used to determine 1) the association between helminth infections (either all helminths or individual species) with malaria parasites (either all or individual species); 2) the effect of decreasing helminth infection on plasmodia parasitaemia and incidence of malaria cases and 3) the effect of decreasing helminth infection on immunological responses to malaria antigen. All analyses will be adjusted for confounders such as socioeconomic status, body mass index, age and sex. During the study any other confounders that are identified will be used in our analyses.

### Power calculation

Unpublished microscopy data from the area showed the combined prevalence of *P. falciparum *and *P. vivax *was 12% in the target population and the helminth infection (*A. lumbricoides*, Hookworm, *T. trichiura*) in the population was 60%. As study activities such as active follow-up and prompt treatment will have an effect on the prevalence, we expect the malaria prevalence to decrease to an estimated 6%. Based on these findings a power calculation (given an alpha of 0.05 and a power of 0.80) gave a sample size of 749 study subjects needed, in order to detect a 50% reduction or increase in the prevalence of plasmodia after 2 years of anthelminthic treatment. The effect of treatment will presumably be present in helminth carriers only and as an estimated 60% will be carriers of helminths during study period, giving an estimated 1248 subjects in each study arm will be needed. Allowing for an estimated 20% lost to follow-up due to movement and refusal, we would need to include 1495 participants in each arm.

### Ethical consideration and trial registration, information, recruitment and consent

This study was approved by The Ethical Committee of Faculty of Medicine, University of Indonesia, ref: 194/PT02.FK/Etik/2006 and registered as clinical trial ref: ISRCTN83830814 and has been filed by ethics committee of the Leiden University Medical Center. The study is reported in accordance with the CONSORT guidelines for cluster-randomized studies [49].

The regional health authorities in Ende, the regional capital, were informed of the study and gave their agreement and support. Socialization took place over a two year period prior to the study involving staff at the public health centre (Puskesmas) and 50 community workers, including training for follow-up of study subjects keeping the community well-informed and well-engaged. Through many organized sessions, the village heads were involved in passing on information about the study, including the benefits and risks involved. The longitudinal nature was explained and information sheets and consent forms (in Bahasa Indonesia) were distributed. Inhabitants of the area were invited to participate in the study, consented either by written signature or thumb print and were informed that they may withdraw from the study at any time, for any reason and without consequences. For children below 15 years old of age also parent consent was obtained. Probable illness by burden of helminth infection will be taken into account by three study doctors that will be present in the area as well as by collaboration with the Puskesmas. Severe cases will be treated directly. The medical doctors will also give support and treatment to the Puskesmas. After completion of the study the whole population will be adequately treated for helminth infections [32,50].

### Description of the population recruited

**Figure 2:**
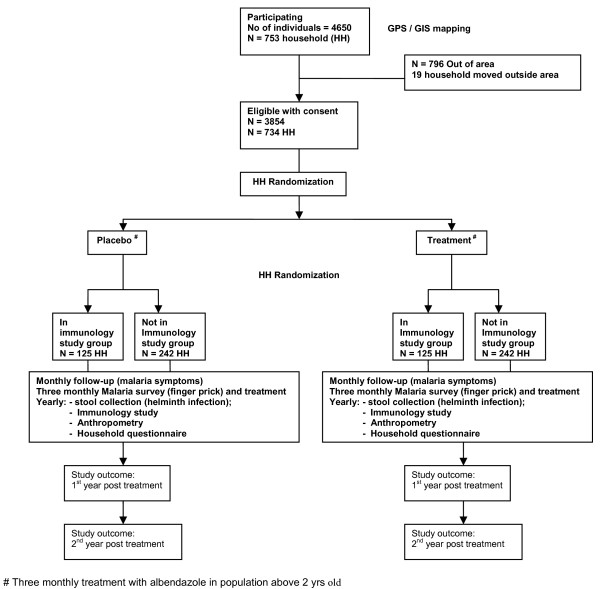
**Profile of the ImmunoSPIN-Malaria sub project **http://immunospin.org

The study has so far provided data that are shown in a flow chart given in Figure 2. During registration (January 2007 - April 2008) a total of 4650 individuals were registered in 753 households. At baseline in (April 2008) 3854 of the registered population are residing in the village in 734 household. For the immunological studies 250 households were randomly selected within 734 registered households, to allow for 1065 eligible individuals (Figure 2).

**Figure 3:**
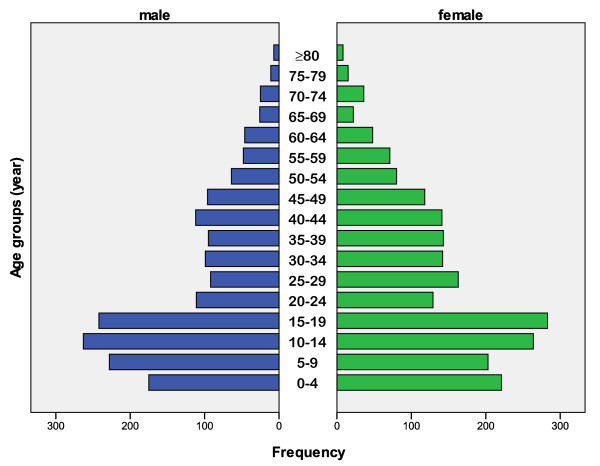
**Age pyramid of individuals living in Nangapanda, Flores**.

Table 1 shows age-stratified migration patterns. Most migration is for seeking employment or education opportunities and most are male. The median age of the residents is 20 years while this is 19 for those who move out of the area. The age pyramid is shown in Figure 3 and is typical for low to middle income countries [51].

**Table 1:** Age distribution of the study population who have stayed or moved out of the study area in 16 months of period*

Age group (years)	Staying			Moved		
	
	Male (n = 1740)	Female (n = 2087)	Total (n = 3827)	Male (n = 431)	Female (n = 365)	Total (n = 796)
**0-4**	175 (10%)	221 (10.6%)	396 (10.3%)	10 (2.3%)	11 (3%)	21 (2.6%)
**5-14**	491 (28.2%)	467 (22.4%)	958 (25%)	49 (11.4%)	47 (12.9%)	96 (12.1%)
**15-24**	353 (20.3%)	412 (19.7%)	765 (20%)	248 (57.5%)	224 (61.4%)	472 (59.3%)
**25-34**	191 (11%)	305 (14.6%)	496 (13%)	71 (16.5%)	53 (14.5%)	124 (15.6%)
**35-44**	207 (11.9%)	284 (13.6%)	491 (12.8%)	37 (8.6%)	15 (4.1%)	52 (6.5%)
**45-54**	160 (9.2%)	198 (9.5%)	358 (9.4%)	8 (1.8%)	8 (2.2%)	16 (2%)
**55-64**	94 (5,4%)	119 (5.7%)	213 (5.6%)	2 (0.5%)	3 (0.9%)	5 (0.6%)
**> = 65**	69 (4%)	81 (3.9%)	150 (3.9%)	6 (1.4%)	4 (1%)	10 (1.3%)

* Data from 4623 of 4650, because 27 individuals was not known for their age or birthday.

**Figure 4:**
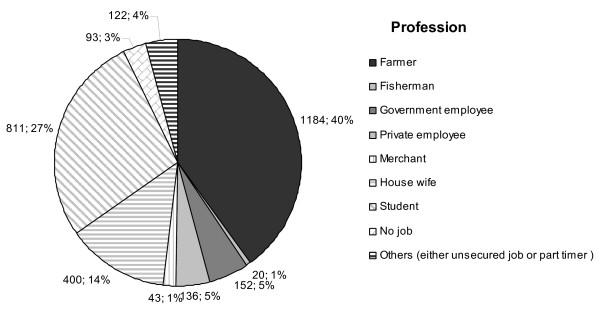
**Job distribution**. At baseline, profession was assessed for everyone above 15 years of age in the study area (n = 2961).

**Figure 5:**
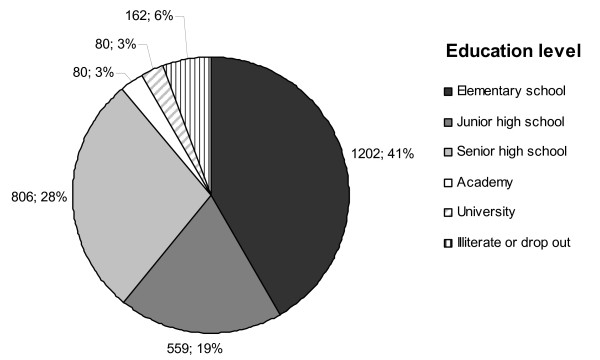
**Education level**. At baseline, education was assessed for everyone above 15 years of age in the study area (n = 2961).

The traditional source of income in the area is farming and fishing while some individuals engage in jobs at government offices with a few in the private sector (Figure 4). The education level of the majority over 15 years of age is elementary school (40.6%) followed by senior high school (27,2%), and junior high school (18.9%) while 5.4% has college or University degrees. Around 5.5% is illiterate, either not educated at all or dropped out from elementary school (Figure 5).

## Discussion

Since this large community-based trial provides an important possibility to undertake a series of evaluations on the effect of helminth infections on malaria as well as the control of helminth and malaria at the community level, our study could help develop evidence-based policymaking. This study is unique in that it will provide data on anthelminthic treatment efficacy and effectiveness in a defined large population in a developing country. In conclusion the ImmunoSPIN helminth-malaria study is the first and currently the only longitudinal study of helminth and malaria co-infections in Indonesia. The study has received enthusiastic support from the authorities in Ende. At the same time, the study facilitates the transfer of state of the art technologies in immunology, molecular biology, epidemiology and statistics to Indonesia.

## Abbreviations

IgE: immunoglobulin E; IL: interleukin;

## Competing interests

The authors declare that they have no competing interests.

## Authors' contributions

AEW Medical doctor in charge of the field study, involve in setting up, supervising gathering of data, clinical care, and follow up of the study population

MAP Medical doctor in charge of the field study, involve in setting up, supervising gathering of data, clinical care, and follow up of the study population

FH Medical doctor in charge of the field study, involve in setting up, supervising gathering of data, clinical care, and follow up of the study population

LW Medical doctor in charge of the field study, involve in setting up, supervising gathering of data, clinical care, and follow up of the study population

BL Medical doctor who is the advisor on databases, epidemiological and statistical aspects of the study

IA Medical doctor who is the advisor on databases, epidemiological and statistical aspects of the study

HWU Statistician who is developing methods to analyze the complex data generated during the lifetime of the project

HW Parasitologist and field study expert who is in charge of the process of data selection, storage, safeguarding randomization, and privacy of the study subjects

YD Medical doctor who advises on the immunological aspects of the study

SW Medical doctor who is supervising of study set up

IS Medical doctor who is a specialist on malaria and advises on clinical malaria and in the study

LM Immunoepidemiologist who is advising on databases maintenance, epidemiological, statistical, and immunological aspects of the study

AJFL Immunologist who specializes in malaria immunology and advises on malaria responses in the study

JJV Molecular parasitologist who is involved in the molecular diagnosis of parasitic infections

ES Immunoparasitologist who is involved in coordinating the study and advising on parasitological and immunological aspects of the study

MY Immunologist who has developed the study and is the Dutch coordinator of the ImmunoSPIN program

TS Parasitologist who has developed the study and is the Indonesian coordinator of the ImmunoSPIN program

All authors read and approved the final paper.

## Pre-publication history

The pre-publication history for this paper can be accessed here:

http://www.biomedcentral.com/1471-2334/10/77/prepub
